# Septin4 promotes cardiomyocytes apoptosis by enhancing the VHL-mediated degradation of HIF-1α

**DOI:** 10.1038/s41420-021-00563-4

**Published:** 2021-07-06

**Authors:** Shaojun Wu, Ying Zhang, Shilong You, Saien Lu, Naijin Zhang, Yingxian Sun

**Affiliations:** Department of Cardiology, the First Hospital of China Medical University, Shenyang, Liaoning PR China

**Keywords:** Apoptosis, Cardiovascular diseases

## Abstract

Septin4, a protein localized at mitochondrion, can promote cells apoptosis mainly by binding XIAP (X-linked inhibitors of apoptosis), however, nothing is known about the role and mechanism of Septin4 in cardiomyocytes apoptosis. Here in the current study, we report that HIF-1α (hypoxia-inducible factor 1 alpha) is a novel interacting protein with Septin4 at Septin4-GTPase domain. In addition, Septin4 enhances the binding between HIF-1α and the E3 ubiquitin ligase VHL (von Hippel-Lindau protein) to down-regulate HIF-1α, and by reducing cardio-protective factor HIF-1α levels, Septin4 aggravated the hypoxia-induced cardiomyocytes apoptosis. We believe these findings will be beneficial to provide effective strategies for clinical treatment of myocardial ischemia and the subsequent injury caused by myocardial hypoxia.

## Introduction

Myocardial ischemia can lead to insufficient oxygen supply to the myocardium, which is also called myocardial hypoxia; myocardial hypoxia can result in cardiomyocytes apoptosis and necrosis [1], which can further develop into myocardial infarction and endanger the life of the patient. HIF-1 plays an important role in mediating cells to adapt to hypoxia, and HIF-1α, the oxygen regulating subunit of HIF-1, is indispensable in this process [2–6]. Under hypoxia, the rapid degradation of HIF-1α in living cells is inhibited, and HIF-1α will subsequently accumulate to a certain level to protect the cardiomyocytes from hypoxia-induced apoptosis mainly by up-regulating the anaerobic process, down-regulating the aerobic process, and restoring the normal delivery of oxygen [7].

Under normoxia, HIF-1α could hardly be detected in cells, which is due to the existence of VHL, a recognizing subunit of an E3 ubiquitin ligase complex that mediates the degradation of HIF-1α through the UPS (ubiquitination-proteasome system) [4–6]. The mechanism has been detailed described that after hydroxylated by prolyl hydroxylase domain enzymes, HIF-1α forms a recognizing site for VHL to bind with, then the binding between VHL and HIF-1α initiate the polyubiquitination and degradation of HIF-1α through UPS pathway [7]. In addition, researchers have found that the VHL-mediated degradation of HIF-1α can be enhanced by some molecules such as VBP1 [8] and SSAT2 [9].

Septin4, a protein localized at mitochondrion, is shown to be a proapoptotic protein mainly by promoting the degradation of XIAP, the only IAP protein that can directly inhibit caspases [10, 11]. Recently, BAX [12] and Bcl-2 (ref. [13]) are also found to be the substrate of Septin4 to promote apoptosis in some tumor cells. However, the role of Septin4 in hypoxia-induced cardiomyocytes and whether there is another novel substrate for Septin4 in cardiomyocytes are not yet known.

Here in this study, we found that the cardio-protective factor HIF-1α is a novel interacting protein with Septin4 via Septin4-GTPase domain in hypoxia-induced cardiomyocytes apoptosis. In addition, although it cannot be categorized as any key enzymes in the UPS, Septin4 aggravated the hypoxia-induced cardiomyocytes by reducing HIF-1α levels through the UPS pathway. In fact, previous studies have reported that Septin4 can promote the ubiquitination and degradation of some proteins by enhancing the interaction between these proteins and some E3 ubiquitin ligases [14, 15]. The current study found the similar mechanism for the first time in cardiomyocytes that Septin4 can enhance the binding between HIF-1α and the E3 ubiquitin ligase VHL, and then Septin4 reduces the expression levels of the cardio-protective factor HIF-1α through UPS pathway. Finally, by reducing HIF-1α levels, Septin4 aggravated the hypoxia-induced cardiomyocytes apoptosis.

## Results

### Septin4 aggravates hypoxia-induced cardiomyocytes apoptosis

**Fig. 1:**
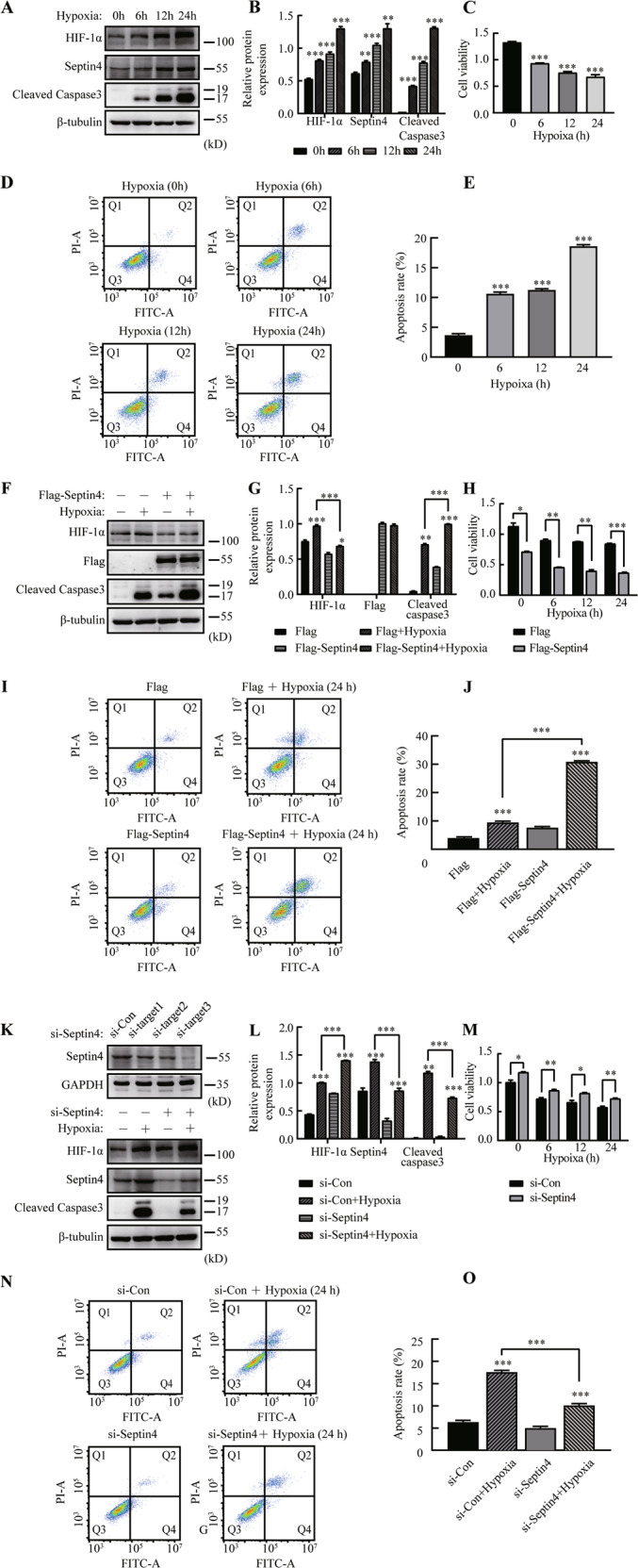
Septin4 aggravates hypoxia-induced cardiomyocytes injury. **A**, **B** Western blot analysis of the expression levels of Septin4, HIF-1α, and cleaved caspases3 with the prolonging of hypoxic time. **C** Cell viability assay of H9c2 cells’ viability with the prolonging of hypoxic time. **D**, **E** Flow cytometry analysis of H9c2 cells’ apoptosis rate with the prolonging of hypoxic time. **F**, **G** Western blot analysis of the expression levels of HIF-1α, Septin4, and cleaved caspases3 in H9c2 cells transfected with vector empty or Flag-Septin4 under hypoxia for 24 h. **H** Cell viability assay of H9c2 cells’ viability after transfected with vector empty or Flag-Septin4 under hypoxia for 24 h. **I**, **J** Flow cytometry analysis of H9c2 cells’ apoptosis rate after transfected with vector empty or Flag-Septin4 under hypoxia for 24 h. **K**, **L** Western blot analysis of the expression levels of HIF-1α, Septin4, and cleaved caspases3 in H9c2 cells transfected with si-Con or si-Septin4 under hypoxia for 24 h. **M** Cell viability assay of H9c2 cells’ viability after transfected with si-Con or si-Septin4 under hypoxia for 24 h. **N**, **O** Flow cytometry analysis of H9c2 cells’ apoptosis rate after transfected with si-Con or si-Septin4 under hypoxia for 24 h. All data are presented as mean ± SD of three repeated experiments; ***<0.001, **<0.01, *<0.05. One-way ANOVA with Tukey’s multiple comparisons test (**B**, **C**, **E**, **G**, **J**, **L**, and **O**); two-way ANOVA with Tukey’s multiple comparisons test (**H**, **M**).

Given that no study has evaluated the effect of hypoxia-induced cardiomyocytes apoptosis on Septin4 expression so far, we first subjected H9c2 cells to hypoxia treatment for 0, 6, 12 and 24 h. Successful establishment of the hypoxic model was confirmed by the observation of an obviously decreased cells viability (Fig. 1C) and a significantly increased cells apoptosis rate (Fig. 1D, E). In addition, by western blot analysis, we found obviously increased expression levels of Septin4 and cleaved caspase3 with the prolonging of hypoxic time (Fig. 1A, B). Thus, Septin4 may play a role in the hypoxia-induced apoptosis, which was discussed in the following experiments on overexpression and knockdown of Septin4 in H9c2 cells.

Cell viability assay and flow cytometry analysis showed that overexpression of Septin4 significantly aggravated hypoxia-induced H9c2 apoptosis (Fig. 1H–J), while knockdown of Septin4 by using the identified most efficacious Septin4 siRNA sequence (Fig. 1K) significantly alleviated hypoxia-induced H9c2 apoptosis (Fig. 1M–O). In addition, overexpression of Septin4 significantly increased apoptosis maker cleaved caspase3 protein levels (Fig. 1F, G), while knockdown of Septin4 significantly decreased cleaved caspase3 levels (Fig. 1K, L). Taken as a whole, these results demonstrated that Septin4 played a negative role in cardiomyocytes survival under hypoxia.

### Septin4 aggravates hypoxia-induced cardiomyocytes apoptosis by down-regulating HIF-1α levels

**Fig. 2:**
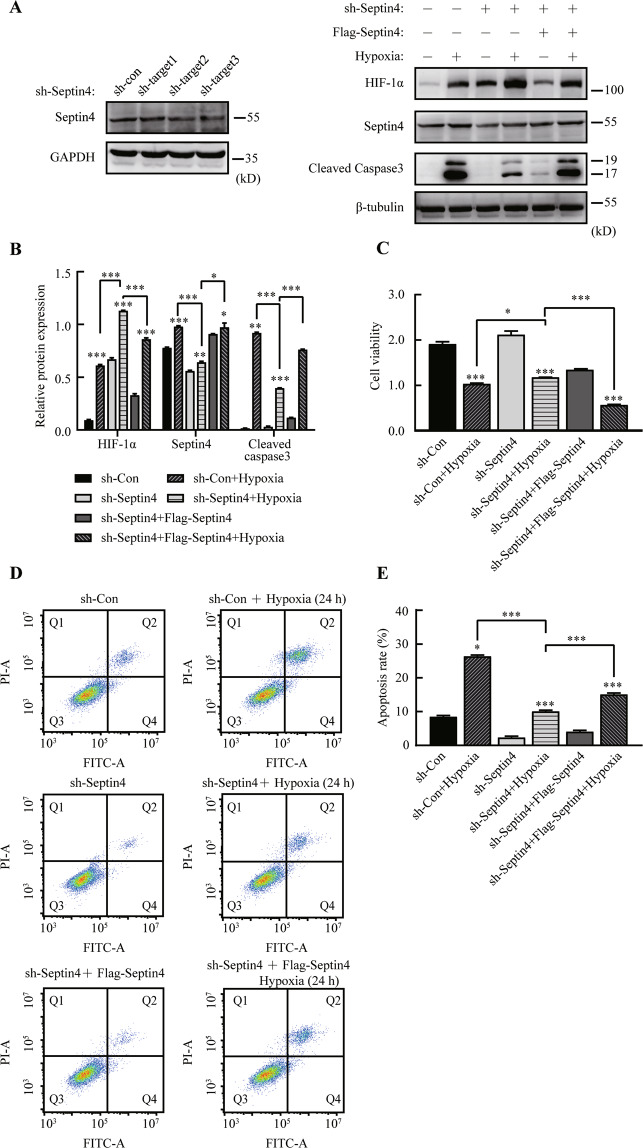
Septin4 aggravates hypoxia-induced cardiomyocytes injury by down-regulating HIF-1α levels. **A**, **B** Western blot analysis of the role of Septin4 in regulating the expression levels of HIF-1α in sh-Septin4 H9c2 cells transfected with vector empty or Flag-Septin4 under hypoxia for 24 h. **C** Cell viability assay of sh-Septin4 H9c2 cells’ viability after transfected with vector empty or Flag-Septin4 under hypoxia for 24 h. **D**, **E** Flow cytometry analysis of sh-Septin4 H9c2 cells’ apoptosis rate after transfected with vector empty or Flag-Septin4 under hypoxia for 24 h. All data are presented as mean ± SD of three repeated experiments; ***<0.001, **<0.01, *<0.05. One-way ANOVA with Tukey’s multiple comparisons test (**B**, **C**, and **E**).

In the experiments on overexpression and knockdown of Septin4 in H9c2 cells, we found that HIF-1α levels decreased after overexpressing Septin4 (Fig. 1F, G) while increased after knocking down Septin4 (Fig. 1K, L). We next explored whether the changes of HIF-1α levels in H9c2 cells was induced by Septin4 by using stable Septin4 knockout H9c2 cell line. We found that HIF-1α levels significantly increased in hypoxic H9c2 cells with stably silenced of Septin4, while markedly decreased after the re-overexpression of Septin4 on the basis of stably silenced of Septin4 (Fig. 2A, B).

At the same time, we observed an opposite expression trend of the apoptosis maker cleaved caspase3 compared to that of HIF-1α in each group (Fig. 2A, B). In addition, cell viability assay (Fig. 2C) and flow cytometry analysis (Fig. 2D, E) showed that the hypoxia-induced apoptosis was relieved with stably silenced of Septin4, while was aggravated with re-overexpression of Septin4 on the basis of stably silenced of Septin4.

These findings suggested that Septin4 aggravated the hypoxia-induced cardiomyocytes apoptosis by down-regulating HIF-1α.

### Septin4 interacts with HIF-1α mainly through its GTPase domain

**Fig. 3:**
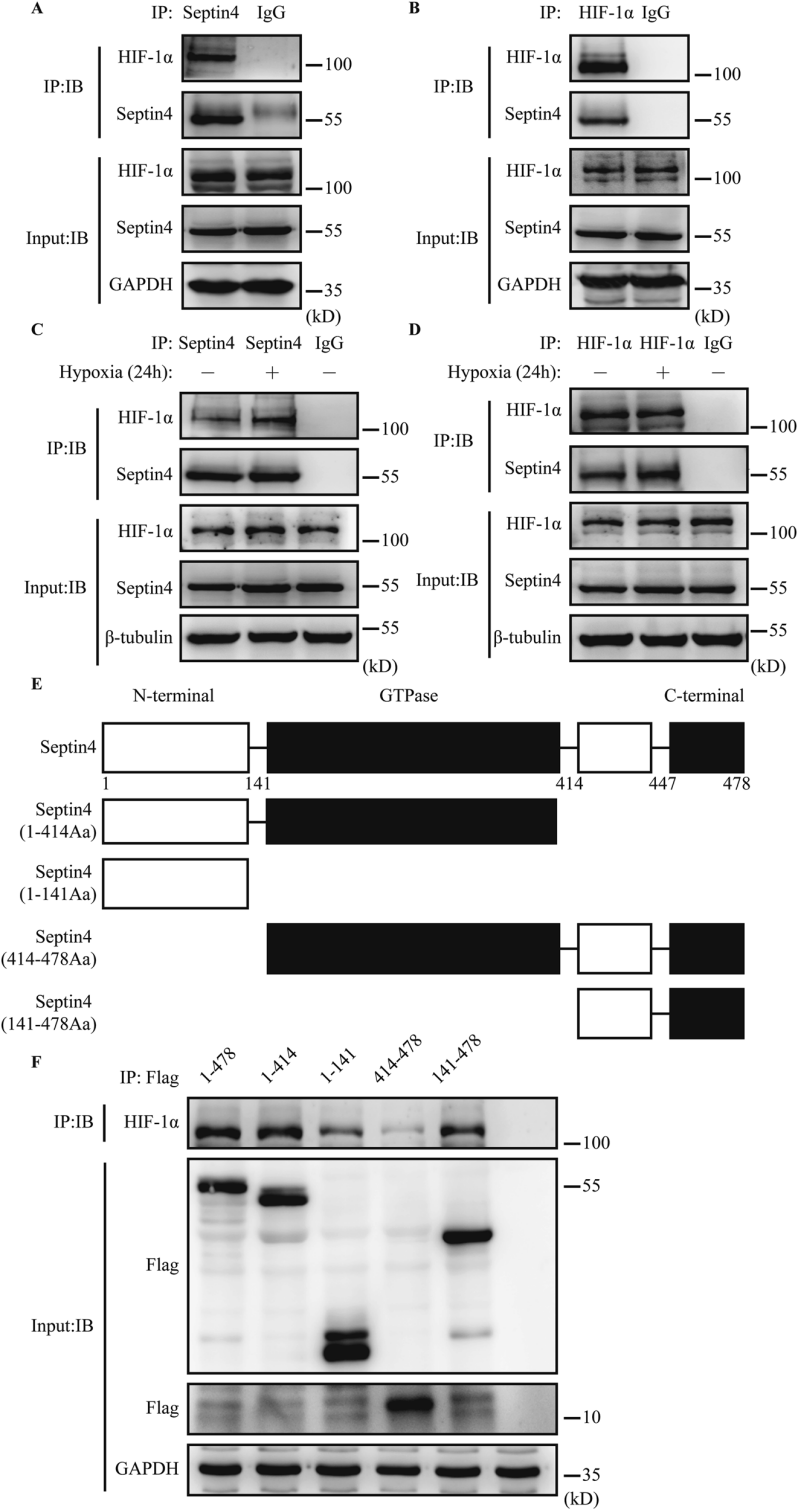
Septin4 interacts with HIF-1α mainly through its GTPase domain. **A**, **B** Co-immunoprecipitation analysis of the endogenous interaction between Septin4 and HIF-1α. **C**, **D** Co-immunoprecipitation analysis of the endogenous interaction between Septin4 and HIF-1α under hypoxia for 24 h. **E**, **F** Co-immunoprecipitation analysis of the interaction between different truncations of Septin4 (exogenously overexpressed) and HIF-1α.

To explore the mechanism of Septin4 in cardiomyocytes apoptosis, we investigated whether Septin4 and HIF-1α actually interact, which then may affect the injury to H9c2 cells. First, results of endogenous co-immunoprecipitation demonstrated that HIF-1α is a novel protein interacting with Septin4 (Fig. 3A, B). Next, we found that under hypoxic stimulation, the binding between Septin4 and HIF-1α was enhanced (Fig. 3C, D). These findings suggested that Septin4 may participate in hypoxia-induced cardiomyocytes injury by interacting with HIF-1α.

Furthermore, to explore which domain of Septin4 bound with HIF-1α, four truncated plasmids of Septin4 were produced according to their functional domains (Fig. 3E). We then overexpressed full-length Septin4 or various truncated mutants of Septin4 in 293 T cells for co-immunoprecipitation, the results demonstrated that Septin4 mainly interacted with HIF-1α via its GTPase domain (Fig. 3F).

### Septin4 mediates the proteasome degradation of HIF-1α

**Fig. 4:**
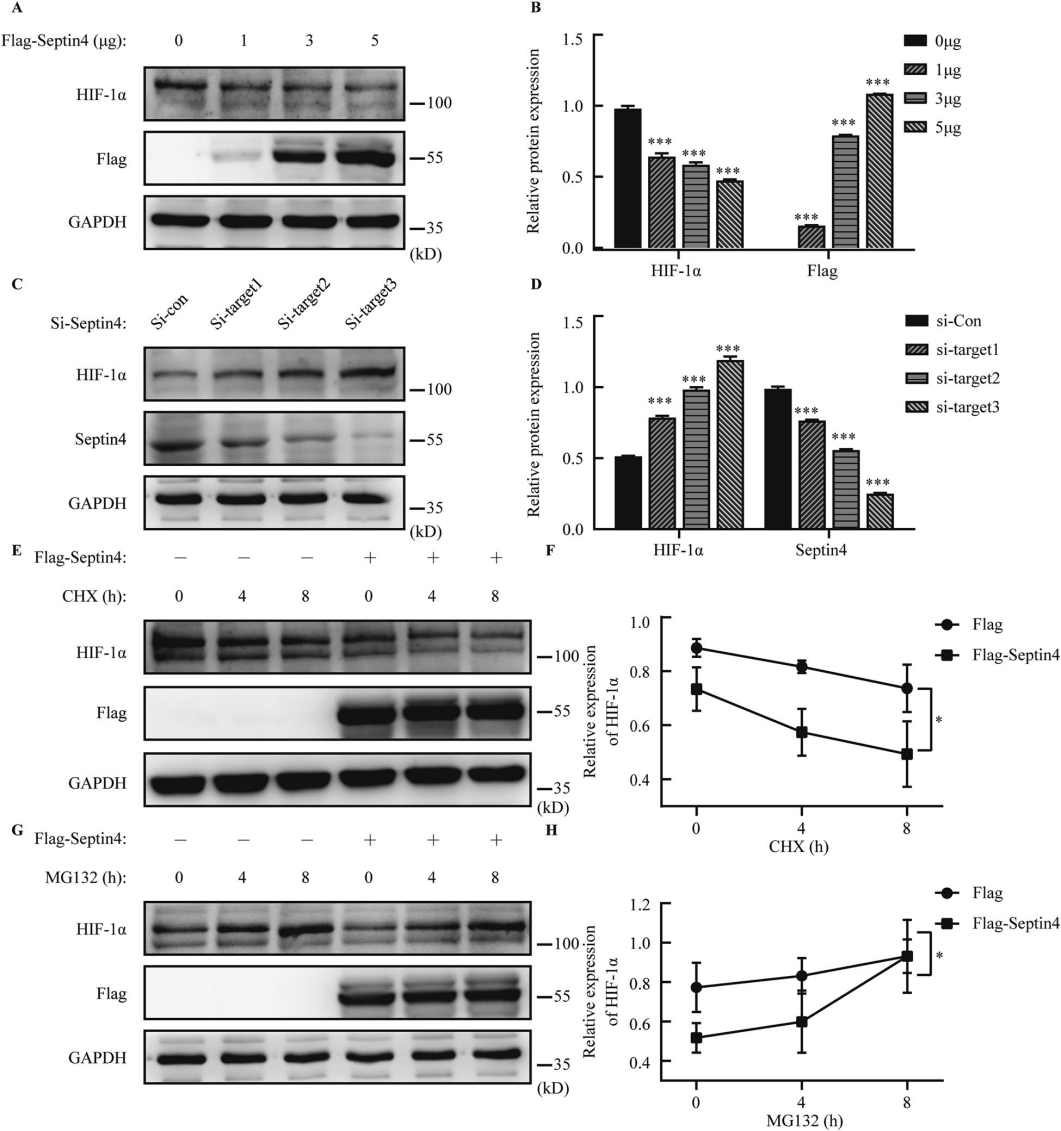
Septin4 mediates the proteasome degradation of HIF-1α. **A**, **B** Western blot analysis of the gradient overexpression of Septin4 and the changes of HIF-1α protein expression levels. **C**, **D** Western blot analysis of the knockdown of Septin4 with three different sequences and the changes of HIF-1α protein expression levels **E**, **F** Western blot analysis of the expression levels of HIF-1α under the treatment of CHX for different hours in H9c2 cells transfected with vector empty or Flag-Septin4. **G**, **H** Western blot analysis of the expression levels of HIF-1α under the treatment of MG132 for different hours in H9c2 cells transfected with vector empty or Flag-Septin4. All data are presented as mean ± SD of three repeated experiments; ***<0.001, **<0.01, *<0.05. One-way ANOVA with Tukey’s multiple comparisons test (**B**, **D**). Two-way ANOVA with Tukey’s multiple comparisons test (**F**, **H**).

Now, we have confirmed that Septin4 and HIF-1α actually bind with each other, so we further explored how Septin4 can reduce HIF-1α levels. First, Flag-Septin4 was transfected in H9c2 cells with an increasing amount. We found that the expression of Septin4 increased while the expression of HIF-1α decreased (Fig. 4A, B). Second, the knockdown of Septin4 with the three Septin4 siRNA sequences resulted in an obviously decreased expression of Septin4 but a significantly increased expression of HIF-1α (Fig. 4C, D). These two findings suggested that Septin4 may mediate the degradation of HIF-1α in some way. Therefore, we further investigated whether Septin4 affected the stability of HIF-1α by the use of protein synthesis inhibitor CHX to inhibit the transcription activity or proteasome inhibitor MG132 to inhibit the proteasome degradation activity.

In the CHX assay, transfection of Flag-Septin4 in H9c2 cells led to an obvious reduction in the expression of endogenous HIF-1α compared to the empty vector group (Fig. 4E, F), while in the MG132 assay with Flag-Septin4 transfected in H9c2 cells, the accumulation of HIF-1α was more marked and faster than the empty vector group (Fig. 4G, H), suggesting that Septin4 reduced the expression of HIF-1α by the proteasome degradation pathway.

### Septin4 mediates the ubiquitination of HIF-1α

**Fig. 5:**
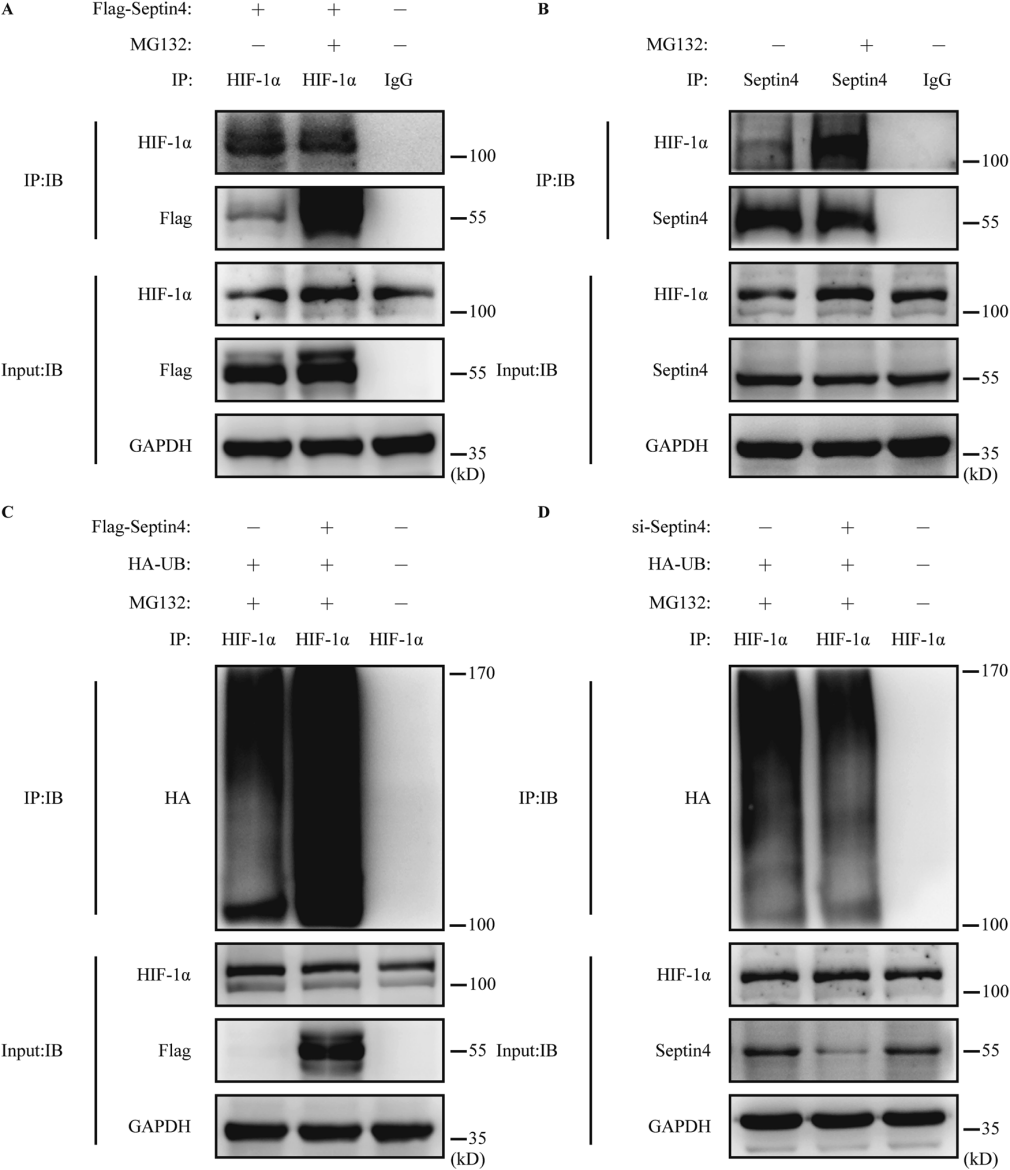
Septin4 mediates the polyubiquitination of HIF-1α. **A** Co-immunoprecipitation analysis of the interaction between exogenous Septin4 and endogenous HIF-1α under the treatment of MG132 for 8 h. **B** Co-immunoprecipitation analysis of the interaction between endogenous Septin4 and endogenous HIF-1α under the treatment of MG132 for 8 h. **C** Co-immunoprecipitation analysis of the role of Septin4 in regulating the ubiquitination levels of HIF-1α in H9c2 cells transfected with vector empty or Flag-Septin4. **D** Co-immunoprecipitation analysis of the role of Septin4 in regulating the ubiquitination levels of HIF-1α in H9c2 cells transfected with si-Con or si-Septin4.

We have confirmed that Septin4 regulates the stability of HIF-1α through the proteasome pathway, so we further explored whether Septin4 can promote the ubiquitination levels of HIF-1α. First, we transfected Flag-Septin4 in H9c2 cells whether followed by the treatment of MG132 or not, and found that the interaction between Septin4 and HIF-1α was enhanced by MG132 (Fig. 5A). The results of endogenous co-immunoprecipitation assay also showed a MG132-enhanced interaction between Septin4 and HIF-1α (Fig. 5B).

Furthermore, after co-transfecting Flag-Septin4 and HA-UB in H9c2 cells followed by the treatment of MG132, we found that Septin4 significantly increased HIF-1α ubiquitination levels by co-immunoprecipitation assay (Fig. 5C). Moreover, Septin4 silencing was found to reduce HIF-1α ubiquitination levels in Septin4 knockdown H9c2 cells after overexpression of HA-UB (Fig. 5D). These results suggested that Septin4-mediated degradation of HIF-1α by the proteasome pathway was dependent on the increased ubiquitination levels of HIF-1α.

### Down-regulation of HIF-1α by Septin4 through ubiquitination-proteasome pathway requires VHL

Actually, Septin4 cannot be categorized as any key enzymes in the ubiquitination-proteasome pathway, but how does it promote the ubiquitination levels of HIF-1α? Therefore, we further explored whether Septin4 can reduce HIF-1α levels by enhancing the effect of some molecules that mediates the UPS degradation of HIF-1α. So we bought HIF-PHDs inhibitor BAY85-3934, which can inhibit the binding between VHL and HIF-1α and then clarified whether Septin4 mediates the ubiquitin-proteasome degradation of HIF-1α via VHL.

**Fig. 6:**
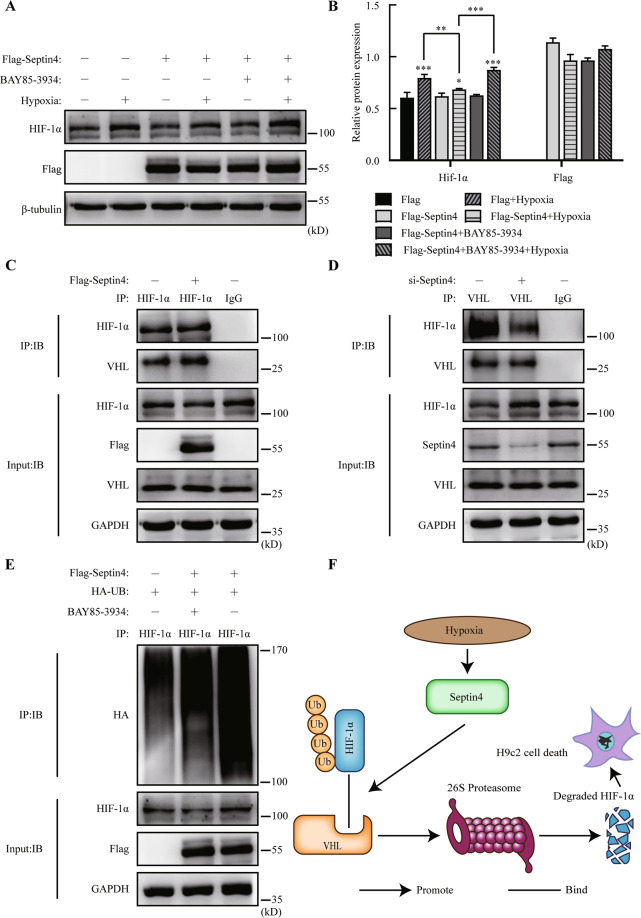
Down-regulation of HIF-1α by Septin4 through ubiquitination-proteasome pathway requires VHL. **A**, **B** Western blot analysis of the expression levels of HIF-1α rescued by BAY85-3934 in H9c2 cells transfected with vector empty or Flag-Septin4. **C** Co-immunoprecipitation analysis of the role of Septin4 in regulating the endogenous interaction between VHL and HIF-1α in H9c2 cells transfected with vector empty or Flag-Septin4. **D** Co-immunoprecipitation analysis of the role of Septin4 in regulating the endogenous interaction between VHL and HIF-1α in H9c2 cells transfected with si-Con or si-Septin4. **E** Co-immunoprecipitation analysis of BAY85-3934’s effect on the necessity of Septin4 regulating the ubiquitination levels of HIF-1α in H9c2 cells transfected with vector empty or Flag-Septin4. **F** Proposed working mode of Septin4 as a negative regulator of HIF-1α by targeting VHL-mediated HIF-1α ubiquitin-proteasome degradation. All data are presented as mean ± SD of three repeated experiments; ***<0.001, **<0.01, *<0.05. One-way ANOVA with Tukey’s multiple comparisons test (**B**).

First, we evaluated the effect of Septin4 and BAY85-3934 on HIF-1α in hypoxic H9c2 cells and found that the expression levels of HIF-1α significantly decreased under hypoxia after the overexpression of Septin4, while BAY85-3934 can rescue this trend (Fig. 6A, B). Second, to explain the phenomenon, we then transfected Flag-Septin4 in H9c2 cells and found that the interaction between HIF-1α and VHL was enhanced (Fig. 6C) while Septin4 silencing was found to reduce the interaction between the two molecules. (Fig. 6D). Lastly, after co-transfecting Flag-Septin4 and HA-UB in H9c2 cells, we found that BAY85-3934 significantly decreased the ubiquitination levels of HIF-1α that was enhanced by Septin4 (Fig. 6E).

These results confirmed that down-regulation of HIF-1α by Septin4 through ubiquitination-proteasome pathway requires VHL, and by reducing HIF-1α levels, Septin4 finally aggravated hypoxia-induced cardiomyocytes apoptosis (Fig. 6F).

## Discussion

For the first time, this study explored the role and mechanism of Septin4 in the hypoxia-induced apoptosis of cardiomyocytes. We showed that the cardio-protective factor HIF-1α was a novel interacting protein with Septin4 via the Septin4-GTPase domain. We also found that Septin4 enhanced the interaction between HIF-1α and the E3 ubiquitin ligase VHL, and thus down-regulated the expression levels of HIF-1α through the ubiquitin-proteasome pathway. In addition, by reducing HIF-1α levels, Septin4 aggravated the hypoxia-induced cardiomyocytes apoptosis.

Previous researches have reported the role of Septin4 in many cancer cells, including non–small-cell lung cancer cell [16], colon cancer cells [12], hepatocellular carcinoma cells [17, 18], malignant melanoma cells [13], neuroblastoma cells [19], myeloid leukemia cells [20, 21], and without exception, Septin4 promotes the apoptosis of all these cells and thus plays a role in cancer suppressing. In addition, Septin4 can also inhibit the growth of hepatic stellate cells and negatively regulates the hepatic fibrosis through TLR4/TGF-β [22] or PI_3_K/Akt pathway [23]. For stem cells such as hair follicle stem cells and intestinal stem cells, they show resistance to apoptosis and have higher levels in Septin4/ARTS^−/−^ mice [24, 25]. We previously reported that Septin4 can aggravate the oxidative stress-induced human umbilical vein endothelial cells injury and thus may be a treatment target of some cardiovascular diseases like atherosclerosis [26, 27]. In the current study, Septin4 acted as a risk factor in cardiomyocyte apoptosis, overexpression of Septin4 aggravated hypoxia-induced cardiomyocyte apoptosis while knocking down Septin4 reduced this apoptosis.

HIF-1α plays a really important role in protecting cardiomyocytes from ischemic injury by increasing expression of its downstream target genes which are involved in angiogenesis, vascular reactivity and glucose metabolism [7, 28], For example, neovascularization stimulated by expression of VEGF (vascular endothelial growth factor) is induced by HIF-1α-mediated transcription of VEGF gene under hypoxia [29]. In addition, injection of AdCA5, an adenovirus providing an active form of HIF-1α shows improved vascular remodeling and angiogenic responses [30, 31]. On the contrary, down-regulation of HIF-1α will show more severe ischemic myocardial injury. A study shows that diallyl trisulfide can decrease ischemic myocardial injury both in rats and in H9c2 cardiomyocytes; however, while the AMPK/AKT/GSK-3β/HIF-1α signaling is inhibited, the cardio-protective effect of diallyl trisulfide is attenuated [32]. Another study shows that HIF-1α silencing decreases cardiotrophin-1 levels and then increases the apoptosis of cardiomyocytes to hypoxia [33]. The current study found that Septin4 can bind to HIF-1α through Septin4-GTPase domain, and just by reducing HIF-1α levels, Septin4 aggravated hypoxia-induced cardiomyocyte apoptosis. Mechanistically, Septin4 enhanced the interaction between HIF-1α and VHL, a classic E3 ubiquitin ligase enzyme that mediates HIF-1α degradation, and then Septin4 promoted the ubiquitination and degradation of HIF-1α. In addition, after the stimulation of BAY85-3934 that can inhibit the binding of VHL to HIF-1α, the ubiquitination and degradation of HIF-1α enhanced by Septin4 was reduced. To sum up, Septin4 aggravated hypoxia-induced cardiomyocytes apoptosis by promoting the VHL-mediated ubiquitination and degradation of the cardio-protective factor HIF-1α.

Contrary to the finding in the current study that Septin4 can promote apoptosis by reducing HIF-1α levels in cardiomyocytes, it has been reported that other proteins in the Septin family like Septin9, can promote tumorigenesis by stabilizing HIF-1α levels in human prostate cancer cells [34, 35]. This is not surprising and we can explain it from the following ways. First, it’s normal that the functions of Septin4 and Septin9 are not exactly the same because they belong to different Septin subgroups. Second, although Septin proteins are all important in regulating cytokinesis in yeast, their contributions to animal cells are now considered to depend on cell type [36]. Third, this two previous studies focus on the competitive effect of Septin9 and RACK1 (receptor of activated protein C kinase 1) to bind HIF-1α while our team mainly studied the mobilizing effect of Septin4 on promoting the binding between VHL and HIF-1α, and Septin4 may also compete with RACK1, but mobilizing VHL is dominant in the current study.

Intriguingly, we may also explain the results of this study that Septin4 aggravated the hypoxia-induced cardiomyocytes apoptosis by reducing HIF-1α levels from the mitochondrion perspective, especially because mitochondrion is involved in cardiomyocytes apoptosis and necrosis in the ischemic heart [1]. Septin 4 is considered to be related to the promotion of MOMP (mitochondrial outer membrane permeabilization) in many ways [14] while HIF-1α is considered to inhibit MOMP [37], and MOMP plays the role in committing cells to die [38]. Therefore, we speculate whether Septin 4 increases cardiomyocytes apoptosis by the increased MOMP which is induced by the reduction of HIF-1α expression. However, this speculation remains to be explored in our future work.

In summary, we have confirmed for the first time that the cardio-protective factor HIF-1α was a new binding protein of Septin4 and Septin4 bound to HIF-1α through the Septin4-GTPase domain. In addition, we also described a mechanism here by which Septin4 aggravated the hypoxia-induced cardiomyocytes apoptosis through the enhanced VHL-mediated degradation of HIF-1α. These findings may provide a new theoretical basis for the treatment of myocardial ischemia.

## Materials and methods

### Cell culture and hypoxia treatment

The rat cardiomyocyte H9c2 cell line and the human embryonic kidney 293 T cell line were procured from the Chinese Academy of Sciences (Shanghai, China) and cultured in DMEM supplemented with 10% FBS (HyClone). All cells were situated in an incubator containing 5% CO_2_ and 95% air at 37 °C. To establish the hypoxia model, H9c2 cells were cultured with serum-free DMEM in an incubator containing 94% N_2_, 5% CO_2_, and 1% O_2_ for 24 h unless otherwise noted.

### Plasmids construction and transfection

Flag-Septin4 (full length) and HA-UB were purchased from GeneChem (China). Four truncated Septin4 plasmids containing different domains with a Flag-tag were obtained: N-terminal and GTPase domains; N-terminal domain; C-terminal domain; C-terminal and GTPase domains. Lipofectamine 3000 (Invitrogen, California, USA) was used for plasmid transfections following the manufacturer’s instructions.

### Septin4 knockdown in H9c2 cells

Normal Control and Septin4 shRNAs were obtained from GeneChem (China). Septin4 silencing was performed with lentivirus. To prevent off-target effects, three sequences were employed:

Septin4 shRNA-1: GCGGAAGAGAGGATCATGCAA

Septin4 shRNA-2: CTGCATCAGCGGGTCAACATT

Septin4 shRNA-3: TGGCCTGAATCGCAAGAACAT

Control and Septin4 siRNAs were obtained from RIBOBIO (China). Septin4 silencing was performed with jetPRIME transfection reagent from PolyPlus (France). To prevent off-target effects, three sequences were employed:

Septin4 siRNA-1: CGGTGGATATAGAAGAGAA

Septin4 siRNA-2: CTATCAGTTCCCAGATTGT

Septin4 siRNA-3: ATGCAAACCGTGGAGATTA

The efficiency of Septin4 knockdown by shRNA and siRNA was confirmed by Western blot analysis.

### Antibodies and reagents

Polyclonal goat anti-Septin4 (abcam, USA), polyclonal rabbit anti-HIF-1α (abcam), polyclonal rabbit anti-VHL (proteintech, USA), polyclonal rabbit anti-caspase-3 (Cell Signaling Technology, USA), monoclonal mouse anti-HA (Cell Signaling Technology), monoclonal mouse anti-Flag (abcam), monoclonal rabbit anti-β-tubulin (Proteintech), and monoclonal mouse anti-GAPDH (abcam). CHX (A8244) and MG132 (A2585), used in DMSO, were purchased from ApexBio (USA). HIF-PHDs inhibitor BAY85-3934 was also purchased from ApexBio.

### Cell viability assay

Cell viability was assessed with a Cell Counting Kit-8 assay (Dojindo, Kumamoto, Japan). Briefly, H9c2 cells were seeded in 96-well plates (NEST Biotechnology) at a density of 5000 cells/well in DMEM with 10% FBS. After being performed the indicated treatments, cells were incubated with 110 μl of CCK-8 solution (serum-free DMEM/CCK-8 reagent = 10/1) per well for 2 h. Finally, the absorbance value of each well was measured at a wavelength of 450 nm with a Microplate ReaderBio-Rad microplate reader (Model 680; Bio Rad Laboratories, Inc., Hercules, CA, USA).

### Flow cytometry analysis

To detect cell apoptosis, Annexin V-FITC/PI Kit (Beyotime Institute of Biotechnology) were used to stain treated cells according to the manufacturer’s instruction, followed by flow cytometry analysis. Briefly, treated H9c2 cells were collected carefully and resuspended in 500 μl binding buffer containing 3.5 μl Annexin V-FITC and 3.5 μl PI to react with H9c2 cells in the dark for 15 min at RT. Finally, apoptotic cells were identified and quantified with a FACSCalibur flow cytometer.

### Protein preparation

For protein analysis, the H9c2 cells were washed with cold PBS, harvested, and lysed as quickly as possible on ice in cell lysis buffer (50 mM Tris, 137 mM NaCl, 1 mM EDTA, 10 mM NaF, 0.1 mM Na_3_VO_4_, 1% NP-40, 1 mM DTT, 10% glycerol, pH 7.8) with both protease (Roche, Switzerland) and phosphatase inhibitors (Bimake, USA), then centrifuged at 13300 rpm/min for 15 min at 4 °C.

### Co-immunoprecipitation and immunoblotting

The lysates were incubated with specific antibodies (antibody/cell lysate = 1 µg/mg) for 3 h followed by adding 30–35 μl Protein A/G beads (Bimake) and incubating for another 12 h at 4 °C. Next, the lysates including antibodies and beads were washed with cell lysis buffer for three times and resolved by 8% or 12% SDS-PAGE gels and then transferred to the PVDF membranes (Millipore USA). Afterwards, the PVDF membranes were blocked by 5% BSA in TBST for 1 h at RT before incubations with primary antibodies overnight at 4 °C and with secondary antibodies for 1 h at RT the next day. Image J 1.52 v (National Institutes of Health, USA) was used to quantify the immunoreactive bands. The expression levels of target protein were shown as fold changes relative to expression levels of corresponding GAPDH or β-tubulin.

### Statistical analysis

Data are presented as mean ± standard deviation (SD). F-test was performed to evaluate the homogeneity of variance and the normality of data was evaluated by Shapiro–Wilk test. To determine the significance of differences between several groups of one related study factor or two, one-way ANOVA and two-way ANOVA were performed respectively, followed by Tukey’s multiple comparisons test. All data were processed by SPSS 25.0 (SPSSInc, Chicago, USA) and GraphPad Prism 8 for Windows, *p* < 0.05 was considered significant.
